# Cardioprotective Effect of Taxifolin against Isoproterenol-Induced Cardiac Injury through Decreasing Oxidative Stress, Inflammation, and Cell Death, and Activating Nrf2/HO-1 in Mice

**DOI:** 10.3390/biom12111546

**Published:** 2022-10-23

**Authors:** Heba M. Obeidat, Osama Y. Althunibat, Manal A. Alfwuaires, Saleem H. Aladaileh, Abdulmohsen I. Algefare, Afaf F. Almuqati, Fawaz Alasmari, Hammad Khalifeh Aldal’in, Abdulkareem A. Alanezi, Bader Alsuwayt, Mohammad H. Abukhalil

**Affiliations:** 1 Department of Medical Analysis, Princess Aisha Bint Al-Hussein College of Nursing and Health Sciences, Al-Hussein Bin Talal University, Ma’an 71111, Jordan; 2 Department of Biological Sciences, Faculty of Science, King Faisal University, Al-Ahsa 31982, Saudi Arabia; 3 Department of Pharmacy Practice, College of Pharmacy, University of Hafr Al-Batin, Hafr Al-Batin 31991, Saudi Arabia; 4 Department of Pharmaceutical Chemistry, College of Pharmacy, University of Hafr Al-Batin, Hafr Al-Batin 31991, Saudi Arabia; 5 Department of Pharmacology and Toxicology, College of Pharmacy, King Saud University, Riyadh 11451, Saudi Arabia; 6 Department of Medical Support, Al-Karak University College, Al-Balqa Applied University, Al-Karak 19117, Jordan; 7 Department of Pharmaceutics, College of Pharmacy, University of Hafr Al-Batin, Hafr Al-Batin 31991, Saudi Arabia; 8 Department of Biology, College of Science, Al-Hussein Bin Talal University, Ma’an 71111, Jordan

**Keywords:** taxifolin, isoproterenol, inflammation, myocardial injury, Nrf2, oxidative stress

## Abstract

Oxidative stress and inflammation are key components in cardiovascular diseases and heart dysfunction. Herein, we evaluated the protective effects of (+)-taxifolin (TAX), a potent flavonoid with significant antioxidant and anti-inflammatory actions, on myocardial oxidative tissue injury, inflammation, and cell death, using a mouse model of isoproterenol (ISO)-induced acute myocardial injury. Mice were given TAX (25 and 50 mg/kg, orally) for 14 days before receiving two subsequent injections of ISO (100 mg/kg, s.c.) at an interval of 24 h on the 15th and 16th days. The ISO-induced cardiac tissue injury was evidenced by increased serum creatine kinase-MB (CK-MB), cardiac troponin I (cTnI), and lactate dehydrogenase (LDH), along with several histopathological changes. The ISO also induced increased malondialdehyde (MDA) with concomitant declined myocardial glutathione level and antioxidant enzymes activities. Moreover, ISO-induced heart injury was accompained with elevated cardiac NF-κB p65, TNF-α, IL-1β, Bax, and caspase-3, as well as decreased Bcl-2, Nrf2, and HO-1. Remarkably, TAX reduced the severity of cardiac injury, oxidative stress, inflammation, and cell death, while enhancing antioxidants, Bcl-2, and Nrf2/HO-1 signaling in ISO-injected mice. In conclusion, TAX protects against ISO-induced acute myocardial injury via activating the Nrf2/HO-1 signaling pathway and attenuating the oxidative tissue injury and key regulators of inflammatory response and apoptosis. Thus, our findings imply that TAX may constitute a new cardioprotective therapy against acute MI, which undoubtedly deserves further exploration in upcoming human trials.

## 1. Introduction

Cardiovascular diseases (CVDs) remain one of the most significant causes of death globally, with an estimated 32% of all deaths worldwide [1,2]. Myocardial infarction (MI) is triggered by a reduction in myocardial blood supply caused by an abrupt blockage of the coronary arteries [3]. Ischemic heart damage is caused primarily by a sudden lack of oxygen and nutrients, as well as the accumulation of metabolic byproducts, eventually culminating in cell injury and death [4]. Despite the broad availability of therapeutic options, MI is still a leading cause of morbidity and death globally; hence, new therapeutic tools for the prevention/treatment of MI are needed. Isoproterenol (ISO) is a common treatment of bradycardia and heart block by acting as a non-selective beta agonist; however, it can lead to functional and structural changes in the myocardium resembling pathological changes observed in acute MI when given at high doses [5,6]. The molecular mechanism by which ISO-induced myocardial injury is complex and multifactorial, with oxidative stress assumed to be the main fundamental mechanism producing pathologic complications involved in ISO cardiotoxicity [6,7].

Oxidative stress can induce tissue injury and plays a key role in the initiation of pathologic alterations in the myocardium, including inflammation and cell death, which eventually leads to the development of cardiac dysfunction and injury [6,7]. The persistently reactive oxygen species (ROS) generation coupled with the dysfunction of important antioxidant defense mechanisms can provoke myocardial inflammation through activating NF-κB and may trigger the stimulation of pro-apoptotic factors in cardiomyocytes, which mediate caspase-dependent and -independent apoptotic cell death [8]. Therefore, mitigation of oxidative stress and inflammation, and activation of cytoprotective pathways, such as the nuclear factor erythroid 2-related factor 2 (Nrf2) signaling pathway, could be a valuable strategy for the prevention and/or treatment of MI and perhaps other CVDs. Indeed, Nrf2 is an important nuclear transcriptional factor that regulates redox homeostasis by activating a variety of antioxidant and detoxifying enzymes [9]. Several studies have indicated that Nrf2 activation prevented cardiac dysfunction and attenuated oxidative tissue injury, inflammatory responses, hypertrophy, and cell death in ISO-intoxicated animals [10,11,12,13,14,15].

**Figure 1:**
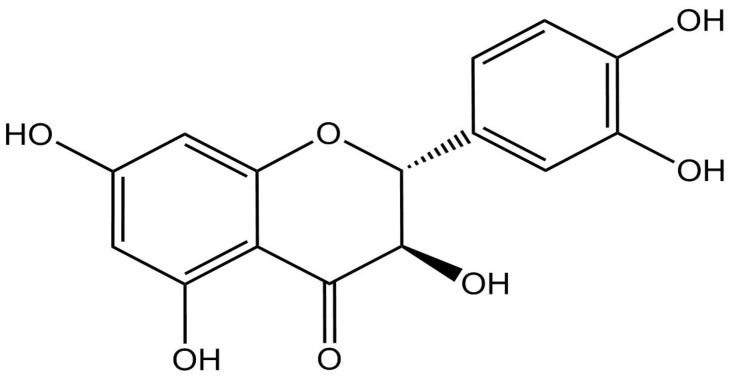
Chemical structure of TAX.

Multiple lines of evidence demonstrate that several natural compounds have shown preventive and therapeutic properties against ISO-induced cardiac injury via modulating ROS overproduction, inflammation, and Nrf2/heme oxygenase-1 (HO-1) signaling [10,11,16,17,18]. (+)-Taxifolin (TAX; C_15_H_12_O_7_, Figure 1) is a natural flavonoid present mainly in olive oil, grapes, and onions, and one which possesses several pharmacological actions, including radical scavenging and anti-inflammatory properties [19,20]. Indeed, TAX was shown to prevent against acrylamide-induced heart damage in rats by decreasing oxidative stress and enhancing antioxidants [21]. Furthermore, TAX protected against renal damage induced by unilateral ureteral obstruction (UUO) in rats via suppressing oxidative tissue injury, inflammation, and fibrosis [22]. Additionally, TAX alleviated myocardial damage triggered by ischemia/reperfusion condition by attenuating oxidative stress and modulating the apoptotic pathway [23]. A recent study showed that TAX prevented cadmium-induced renal damage by modulating redox status, inflammatory response, and apoptotic cell death, as well as by upregulating the Nrf2/HO-1 signaling pathway. An in vitro study showed that TAX protected against oxidative stress-induced retinal pigment epithelium (RPE) cell death through the activation of Nrf2 and its regulated phase II antioxidant enzymes [24]. Moreover, TAX prevented alcohol-induced hepatic apoptosis in mice through modulating oxidative stress, NF-κB-mediated inflammation, and PI3K/Akt signaling pathway [25].

Although multiple therapeutic benefits have been explored, the mechanism behind TAX’s therapeutic impact on ISO-triggered heart damage is yet to be identified. We hypothesized that TAX would be a novel strategy for protecting against the cardiac injury induced by ISO via suppressing myocardial oxidative damage, inflammation, and cell death, and activating Nrf2/HO-1 signaling. Therefore, we looked into the influence of TAX on the markers of oxidative stress, inflammatory response, and apoptosis, as well as the Nrf2/HO-1 signaling pathway in the cardiac tissue of ISO-intoxicated mice. The results of this study may provide a new mechanistic insight into the protective actions of TAX against ISO cardiotoxicity, as well as a potential strategy for CVDs and heart failure prevention.

## 2. Materials and Methods

### 2.1. Reagents and Chemicals

The ISO was procured from Sigma (St. Louis, MO, USA). The TAX was purchased from Biosynth Carbosynth (Berkshire, UK). A Cardiac troponin I (cTnI) ELISA kit was obtained from Kamiya (Tukwila, WA, USA). Kits for creatine kinase-MB (CK-MB) and lactate dehydrogenase (LDH) activities were procured from Spinreact (Girona, Spain). A HO-1 ELISA kit was obtained from MyBioSource (San Diego, CA, USA). The ELISA kits for tumor necrosis factor-α (TNF-α) and interleukin-1β (IL-1β) were supplied by R&D Systems (Minneapolis, MN, USA). Anti-NF-κB p65, anti-caspase-3, and anti-Nrf2 antibodies were obtained from ThermoFisher (Waltham, MA, USA; 1:100 dilution), while anti-Bax and anti-Bcl-2 antibodies were supplied by Abcam (Cambridge, MA, USA; 1:100 dilution).

### 2.2. Animals and Treatment

A mouse model of ISO-triggered acute myocardial injury was used here to examine the cardioprotective action of TAX. Animals were obtained from Jordan University of Science and Technology (JUST), Irbid, Jordan. All animal-related protocols including care, handling, and treatment were verified by an Al-Hussein Bin Talal University panel of animal research and ethics and were in conformity with the National Institute of Health standards (NIH publication No. 85-23, revised 2011). The experiment included 30 Swiss albino mice (25–30 g), which were housed under standard conditions in an environment-controlled room and given free access to food and water. The vehicle solutions of physiological saline and 0.5% DMSO were used to dissolve ISO [12] and TAX [26], respectively.

Following a week of acclimatization, the selected mice were separated into five groups (*n* = 6), as follows. The first group included the control mice which were given 0.5% DMSO for 14 days, using oral gavage, followed by two subcutaneous (s.c) injections of physiological saline on days 15 and 16; the second group (TAX) were given TAX (50 mg/kg) for 14 days followed by two 24-h interval sequential injections (s.c) of physiological saline; groups III (ISO), IV (TAX 25 mg/kg and ISO), and V (TAX 50 mg/kg and ISO) received oral 0.5% DMSO, 25 mg/kg TAX, or 50 mg/kg TAX, respectively, for 14 days before they were injected twice with ISO (100 mg/kg, s.c.) on the 15th and 16th days. The TAX and ISO dosages were chosen based on relevant articles by Algefare [26] and Abukhalil et al. [11], respectively.

At the end of the experiment, anesthesia was induced by injecting mice with ketamine/xylazine (100 mg/kg and 10 mg/kg, respectively, i.p), followed by blood collection via cardiac puncture and immediate heart extraction and processing. The blood samples were left standing until full coagulation occurred, then centrifuged for serum preparation and aliquoted at −20 °C for cardiac marker analysis. The excised hearts were dissected and rinsed in 50 mM (pH 7.0) of cold phosphate buffer saline (PBS). Some heart pieces were preserved in 10% neutral buffered formalin (NBF) for histological evaluation, while others were homogenized in cold PBS (10% *w*/*v*). The homogenate was then processed in refrigerated centrifuge, and the clear supernatant was collected and maintained at −20 °C for further biochemical investigations.

### 2.3. Assessment of Cardiac Biomarkers

A specific ELISA kit was used for measurement of serum cTnI levels, while CK-MB and LDH activities were evaluated spectrophotometrically with commercially available kits. All tests were carried out in accordance with the manufacturers’ instructions.

### 2.4. Investigation of Oxidative Stress Markers and Antioxidants in the Heart

The malondialdehyde (MDA) contents were determined spectrophotometrically using thiobarbituric acid (TBA), as previously described [27]. In brief, the sample MDA was allowed to react with TBA in acidic medium at 95 °C for 30 min to form thiobarbituric acid reactive product, followed by measuring the absorbance of the resultant pink product at 532 nm. The level of protein carbonyl in the myocardial tissues was measured spectrophotometrically, as previously described [28]. The principle of the method depends on protein carbonyl interaction with 2,4-dinitrophenylhydrazine (DNPH) to generate the stable dinitrophenyl (DNP) hydrazine, which was analyzed spectrophotometrically at 375 nm. Nitric oxide (NO) level was determined spectrophotometrically by measurement of its more stable derivatives, nitrate and nitrite, according to the method described previously [29]. Briefly, nitrate was reduced first to nitrite followed by determination of total nitrite by a Griess reaction. This involved the formation of diazotize sulphanilamide from nitrite in an acidic medium, which is then coupled with N-(1–naphthyl) ethylenediamine to produce a chromophoric azo product measured with spectrophotometry at 540 nm.

The contents of reduced glutathione (GSH) were estimated using the method based on the continuous reduction of 5, 5 dithiobis (2-nitrobenzoic acid) by GSH to produce 5-thio-2-nitrobenzoic acid (TNB) which was spectrophotometrically quantified at 412 nm [30]. The superoxide dismutase (SOD) activity was estimated using an assay relying on the ability of the enzyme to inhibit the phenazine methosulphate-mediated reduction of nitro blue tetrazolium dye as previously described [31]. Catalase (CAT) activity was estimated using a method based on its ability to decompose hydrogen peroxide (H_2_O_2_) into water and oxygen [32]. The rate of H_2_O_2_ decomposition was assessed upon measurement of unconverted H_2_O_2_ by reacting it with 3,5-dichloro-2-hydroxybenzenesulfonic acid (DHBS) and 4-aminoantipyrine in the presence of horseradish peroxidase (HRP) to form a product measured via spectrophotometry at 570 nm. Furthermore, a specific ELISA kit was used for the measurement of heart HO-1 content according to the protocol provided.

### 2.5. Investigation of Pro-Inflammatory Cytokines in the Heart

Measurement of cardiac levels of TNF-α and IL-1β were performed by following the manufacturer’s protocol for the specific ELISA kits.

### 2.6. Histopathological and Immunohistochemical Analysis

Following a standard procedure, the NBF preserved cardiac tissue was paraffin embedded, 5-microne slices were prepared, and parts of the deparaffinized sections were stained with hematoxylin and eosin (H&E) [33]. The stained sections were then examined under a light microscope by a histopathologist.

The remaining deparaffinized slices were subjected to immunohistochemistry (IHC) staining [34]. Antigen retrieval was performed by successively treating cardiac tissues with 50 mM citrate buffer (pH 6.8) and 0.3% H_2_O_2_. Then, normal serum was added for 20 min to block nonspecific antigen–antibody binding. After washing the tissues with PBS, anti-NF-κB p65, anti-caspase-3, anti-Bax, anti-Bcl-2, and anti-Nrf2, antibodies were applied overnight at 4 °C. Unbound primary antibodies were washed with PBS followed by incubation of sections with the secondary antibodies. The substrate 3,3-Diaminobenzidine (DAB) was added for color development before the slides were counterstained with Mayer’s hematoxylin and evaluated under a light microscope. The staining labelling indices of the anti-Bax, anti-caspase-3, and anti-NF-κB p65 antibodies were presented as a percentage of positive expression in a total of 1000 cells. By using ImageJ analysis software (NIH, Bethesda, MD, USA), the immunostaining intensity of anti-Bcl-2 and anti-Nrf2 antibodies was determined through the area of positive expression.

### 2.7. Analysis of Data

Data are presented as the mean ± standard error of the mean (SEM). GraphPad Prism 7 software (San Diego, CA, USA) was employed to perform the statistical comparison between groups using one-way analysis of variance (ANOVA) followed by a Tukey’s post-hoc test. Results were considered statistically significant when the *p* value < 0.05.

## 3. Results

### 3.1. TAX Prevents ISO-Triggered Myocardial Injury in Mice

**Figure 2:**
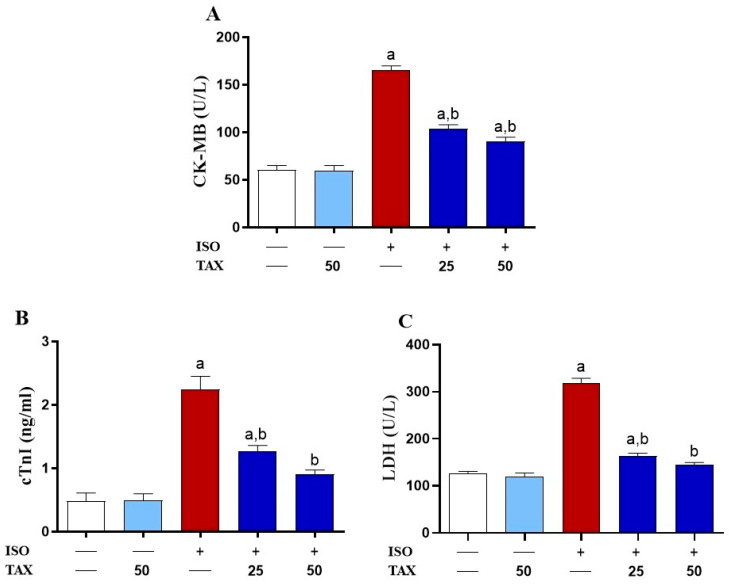
TAX attenuates the ISO-induced cardiac injury. Here, TAX reduced serum levels of (**A**) CK-MB, (**B**) cTnI, and (**C**) LDH in ISO-intoxicated animals. Data are expressed as mean ± SEM, (*n* = 6). Here, a indicates significant (*p* < 0.05) vs. control, while b indicates significant (*p* < 0.05) vs. ISO.

**Figure 3:**
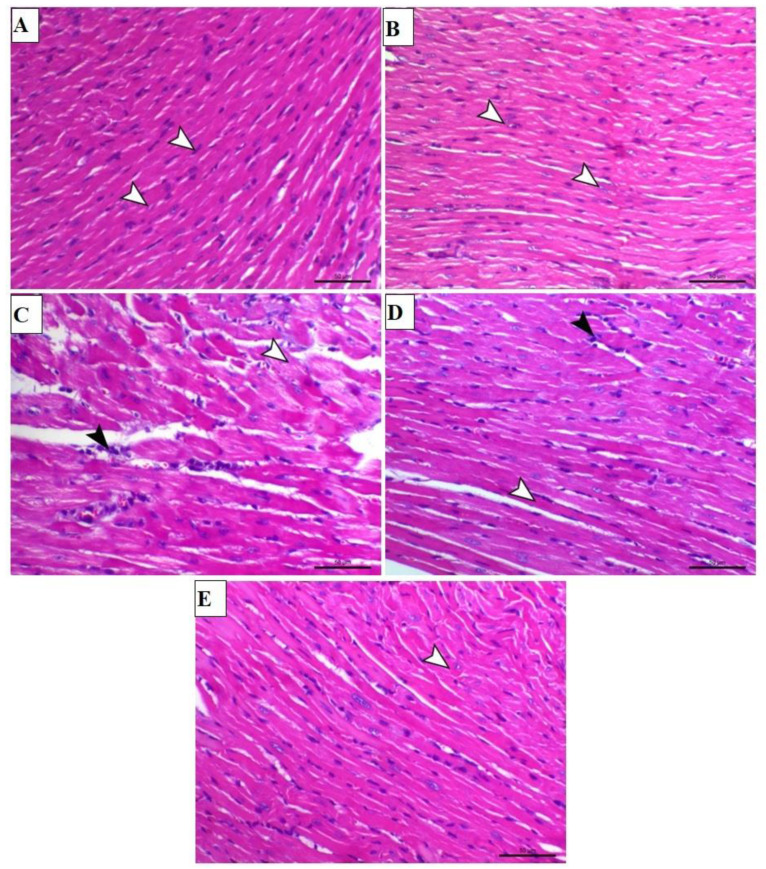
TAX prevents the ISO-induced histopathological damage in the heart. Images of the microscopic field of cardiac sections from (**A**) control and (**B**) TAX-treated mice showing normal branched connected myocardial fibers with a normal central nucleus (arrowheads); (**C**) ISO-treated animals demonstrating marked myolysis (white arrowhead), marked nuclear pyknosis, and infiltration of mononuclear inflammatory cells (black arrowhead); (**D**) ISO-injected mice pre-treated with 25 mg TAX demonstrating a reduction in myocardial degeneration of muscle fibers (white arrowhead) and mild infiltration of mononuclear inflammatory cells (black arrowhead); and (**E**) ISO-injected mice pre-treated with 50 mg TAX demonstrating a noticeable reduction in myocardial degeneration and with mild curving of cardiac muscle fibers (arrowhead) (H&E, X200, Scale bar = 50 µm).

To assess the protective effect of TAX on ISO-induced cardiac injury, we investigated serum cardiac biomarkers levels (Figure 2A–C) and histological changes (Figure 3) in both TAX-treated and untreated mice. The ISO-treated mice showed a significant (*p* < 0.05) increase in serum CK-MB (Figure 2A), cTnI (Figure 2B), and LDH (Figure 2C) as compared to control mice. Pretreatment of ISO-intoxicated mice with TAX markedly (*p* < 0.05) attenuated CK-MB, cTnI, and LDH in serum. Normal mice that received TAX alone had no effects on these cardiac biomarkers in serum.

Furthermore, the effect of TAX on ISO-induced cardiac injury was assessed by evaluating H&E stained heart sections of both TAX-treated and untreated mice. Microscopic observation of heart tissue slides from control and TAX-treated mice demonstrated normal branched connected myocardial fibers with a normal central nucleus (Figure 3A,B). A microscopic field of the hearts of ISO-treated mice demonstrated marked myolysis, marked nuclear pyknosis, and infiltration of mononuclear inflammatory cells (Figure 3C). These histopathological alterations were significantly ameliorated when ISO-intoxicated mice were treated with both doses of TAX (Figure 3D,E).

### 3.2. TAX Attenuates Oxidative Stress and Enhances Antioxidants in Heart of ISO-Injected Mice

**Figure 4:**
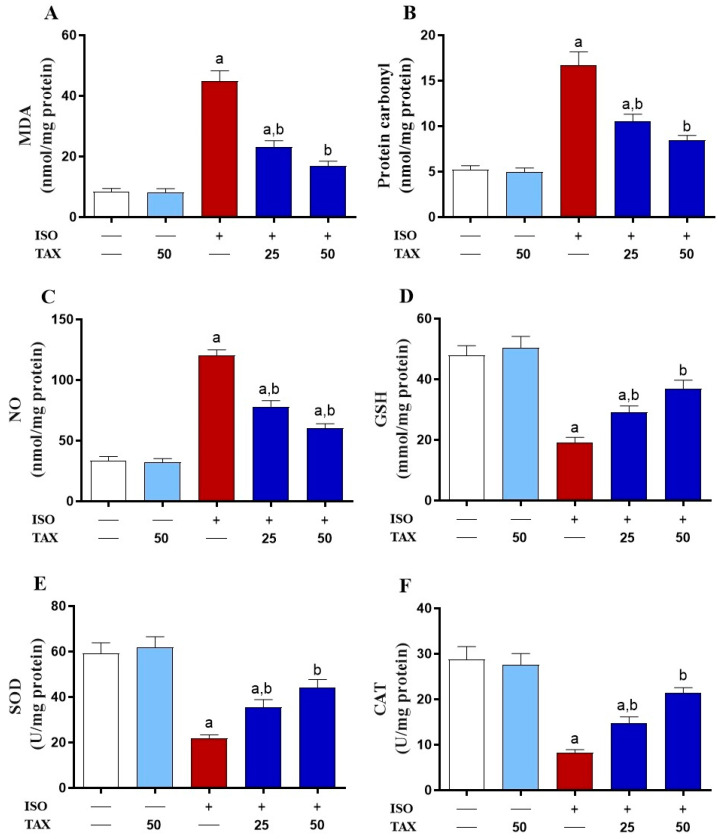
TAX attenuates the ISO-induced myocardial oxidative stress. Pre-treatment with TAX reduced cardiac levels of (**A**) MDA, (**B**) protein carbonyl, (**C**) NO, and elevated (**D**) GSH level, and activities of (**E**) SOD and (**F**) CAT in ISO-injected mice. Data are expressed as mean ± SEM, (*n* = 6). Here, a indicates significant (*p* < 0.05) vs. control, while b indicates significant (*p* < 0.05) vs. ISO.

Hearts of ISO-treated mice showed significantly (*p* < 0.05) increased MDA (Figure 4A), protein carbonyl (Figure 4B), and NO (Figure 3C) contents, along with significantly (*p* < 0.05) decreased GSH (Figure 4D) levels and SOD (Figure 4E) and CAT (Figure 4F) activities as compared to control mice. Pretreatment of ISO-injected mice with TAX resulted in a substantial (*p* < 0.05) reduction in MDA, protein carbonyl, and NO contents and an increase in antioxidants in the heart. The TAX alone did not affect these parameters in healthy mice.

### 3.3. TAX Mitigates Myocardial Inflammation in ISO-Injected Mice

**Figure 5:**
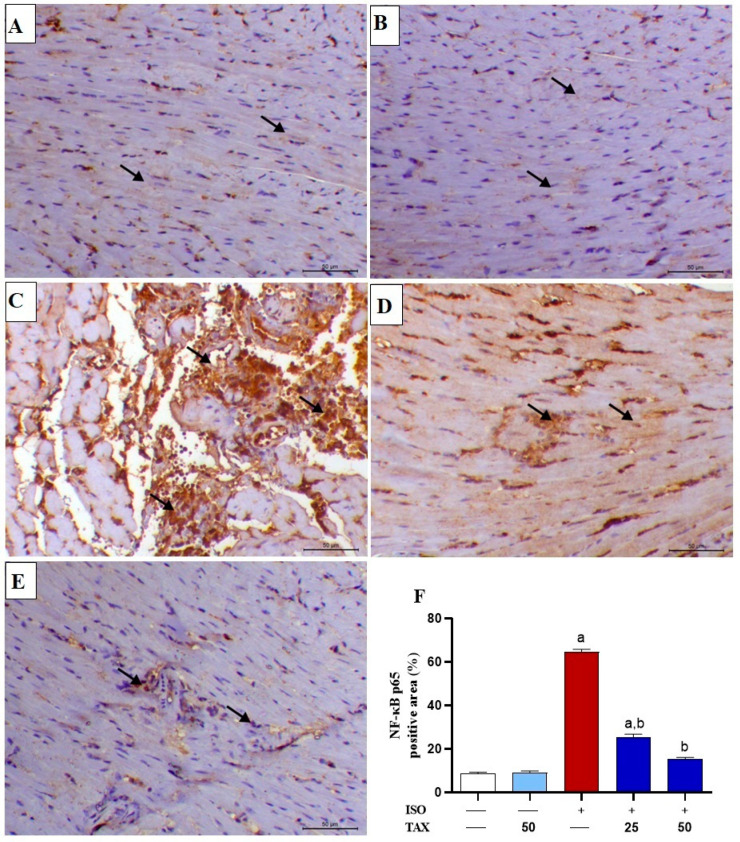
TAX attenuates ISO-induced myocardial NF-κB p65 upregulation. Images of microscopic field of IHC labeled cardiac sections from (**A**) control and (**B**) TAX-treated mice showing mild expression of myocardial NF-κB p65 (arrows designate positive immunostaining within the interstitial cells); (**C**) ISO-injected mice showing a marked increase in both cytoplasmic and nuclear levels of NF-κB p65 antibody within myocytes (arrows designate marked expression within both degenerated fibers and inflammatory cells); (**D**) ISO-administrated mice pre-treated with 25 mg TAX demonstrating a decreased level of the nuclear NF-κB p65 within the myocytes (arrows); and (**E**) ISO-administrated mice pre-treated with 50 mg TAX demonstrating a noticeably decreased expression of the nuclear NF-κB p65 within the myocardial fibers (arrows) (IHC, X200, Scale bar = 50 µm). (**F**) Analysis of the relative intensities of the images of IHC labeled myocardial NF-κB p65 showing a significant increase in ISO-injected mice and a significant reduction in mice pre-treated with both doses of TAX. Data are expressed as mean ± SEM, (*n* = 6). Here, a indicates significant (*p* < 0.05) vs. control, while b indicates significant (*p* < 0.05) vs. ISO.

**Figure 6:**
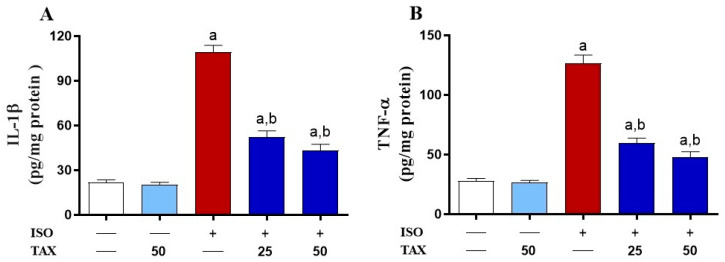
TAX reduced the ISO-induced increased myocardial (**A**) IL-1β and (**B**) TNF-α. Data are expressed as mean ± SEM, (*n* = 6). Here, a indicates significant (*p* < 0.05) vs. control, while b indicates significant (*p* < 0.05) vs. ISO.

The inflammatory response is known to fuel the major pathological processes in ISO-induced cardiac injury. Indeed, ISO induced a considerable (*p* < 0.05) rise in NF-κB p65 expression (Figure 5A–F) and myocardial levels of the pro-inflammatory cytokines, IL-1β (Figure 6A) and TNF-α (Figure 6B). The TAX treatment of ISO-injected mice significantly (*p* < 0.05) attenuated the myocardial contents of NF-κB p65, IL-1β and TNF-α. However, TAX alone did not affect the levels of the above-mentioned inflammatory proteins in the heart.

### 3.4. TAX Prevents Myocardial Apoptosis in ISO-Treated Mice

**Figure 7:**
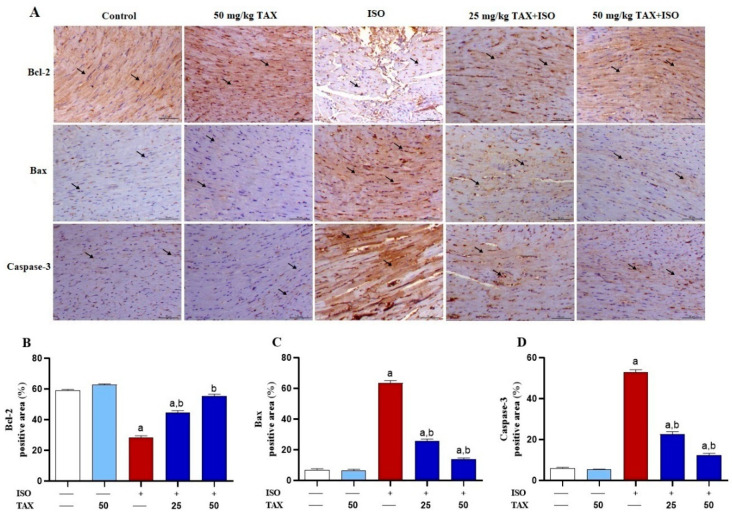
TAX attenuates the ISO-induced myocardial apoptosis in mice. (**A**) Images of the microscopic field of IHC labeled cardiac sections from control and TAX-treated mice, showing obvious expression of Bcl-2 within the myocardial fibers (arrows), along with mild expression of myocardial Bax and caspase-3 (arrows); ISO-injected mice demonstrating a noticeable reduction in the myocardial level of Bcl-2 (arrows), along with remarkably increased myocardial Bax and caspase-3 (arrows); and mice treated with 25 and 50 mg/kg TAX before ISO injection demonstrating a noticeable increase in myocardial expression of Bcl-2 (arrows), along with a marked reduction in the expression of Bax and caspase-3 in the myocardial tissues (arrows). (IHC, X200, Scale bar = 50 µm). (**B**–**D**) Analysis of the relative intensities of the images of IHC-labeled myocardial Bcl-2, Bax, and caspase-3, showing a significantly increased Bcl-2 and decreased Bax and caspase-3 in mice pre-treated with both doses of TAX. Data are expressed as mean ± SEM, (*n* = 6). Here, a indicates significant (*p* < 0.05) vs. control, while b indicates significant (*p* < 0.05) vs. ISO.

To further assess the protective impact of TAX treatment on ISO-triggered myocardial apoptosis, we evaluated Bax, Bcl-2, and caspase-3 expression levels in the myocardial tissues of both TAX-treated and untreated mice by IHC staining. The ISO resulted in a marked (*p* < 0.05) decrease in Bcl-2 expression level (Figure 7A,B), associated with a significant (*p* < 0.05) increase in Bax (Figure 7A,C) and caspase-3 (Figure 7A,D) expression in the heart. The ISO-induced imbalance in the expression of pro-apoptotic and anti-apoptotic proteins were remarkably (*p* < 0.05) attenuated when mice were pre-treated with both doses of TAX. Treatment of normal mice with TAX had no effects on the above-mentioned markers in the heart.

### 3.5. TAX Activates Cardiac Nrf2/HO-1 in ISO-Injected Mice

**Figure 8:**
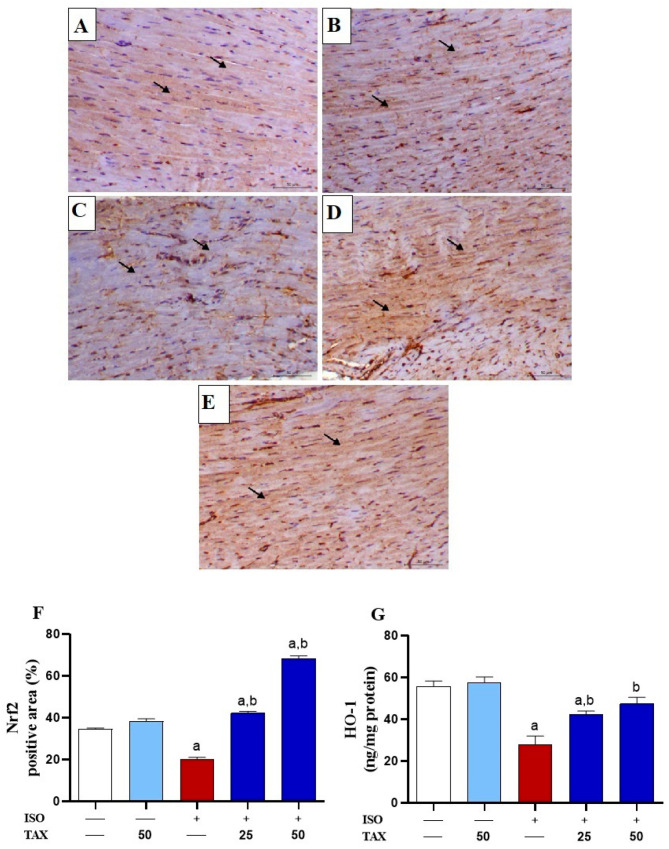
TAX upregulates Nrf2/HO-1 signaling in ISO-administrated mice. Images of the microscopic field of IHC labeled cardiac sections from (**A**) control and (**B**) TAX-treated mice, indicating obvious cytoplasmic immunostaining of Nrf2 within the myocardial fibers (arrows); (**C**) ISO-administered mice showing focal myocardial expression of Nrf2 (arrows); (**D**) ISO-injected mice pre-treated with 25 mg TAX demonstrating increased Nrf2 expression within the myocardial fibers (arrows); and (**E**) ISO-injected mice pre-treated with 50 mg demonstrating a noticeable rise in the myocardial expression of Nrf2 (arrows) (IHC, X200, Scale bar = 50 µm). (**F**) Analysis of the relative intensities of the images of IHC labeled myocardial Nrf2 demonstrating a significant increase in Nrf2 in the myocardium of mice treated with both doses of TAX before ISO injection. (**G**) TAX significantly attenuates myocardial levels of HO-1 in ISO-injected mice. Data are expressed as mean ± SEM, (*n* = 6). Here, a indicates significant (*p* < 0.05) vs. control, while b indicates significant (*p* < 0.05) vs. ISO.

Since Nrf2 is known to suppress surplus ROS and attenuate oxidative tissue injury, changes in its expression (Figure 8A–F) and HO-1 levels (Figure 8G) in the heart of both TAX-treated and untreated mice were estimated. The ISO-treated mice showed significantly (*p* < 0.05) downregulated Nrf2 expression and HO-1 levels in the heart as compared to the control mice. The ISO-induced downregulation of myocardial Nrf2 and HO-1 was significantly (*p* < 0.05) ameliorated by TAX treatment (Figure 8A–G). Treatment of normal mice with TAX alone did not affect Nrf2 and HO-1 levels in the heart.

## 4. Discussion

As CVDs are the leading cause of mortality and morbidity worldwide, there is an urgent need to develop effective interventions to treat CVDs, principally MI. Since oxidative tissue injury and inflammation are key components implicated in the pathological processes of MI, novel compounds with antioxidant and anti-inflammatory effects could exert beneficial actions against the development of cardiovascular dysfunction in MI. Herein, we have assessed the cardioprotective effects of TAX, a flavononol with promising biological activities, on ISO-induced myocardial injury in mice. Our findings demonstrate that TAX prevented ISO-induced oxidative stress, NO formation, inflammation, and apoptosis in the heart, and consequently attenuated myocardial tissue damage.

Repeated injection of ISO offers a non-invasive, low-mortality animal model of acute myocardial injury that mimics acute MI in humans [35]. Consequently, the experimental model of ISO-induced myocardial damage has been employed to assess the effects of various treatments on cardiac dysfunctions. This ISO-induced myocardial damage has been linked to high circulating cardiac biomarkers, as well as the development of numerous histopathological alterations, such as myocyte loss, fibrosis, and inflammatory cell infiltration [11,12,36]. Likewise, our findings demonstrated that ISO-intoxicated mice had substantially higher serum CK-MB, cTnI, and LDH levels when compared to the control group, which was associated with morphological aberrations including necrotic features, misaligned myofibrils, and extensive neutrophil infiltration. It is well known that myocyte apoptosis and necrosis are preceded by membrane disruption, resulting in leakage of cardiac biomarkers and, hence, an increase in their circulating levels [37]. Thus, an increase in serum cardiac biomarkers is considered as a sensitive indicator of altered myocyte permeability under cardiac stress conditions [38]. On the other hand, TAX demonstrated potent cardioprotective actions in ISO-induced mice, which was evidenced by reducing circulating cardiac biomarkers and attenuating histological alterations. Consistently, TAX has shown cardioprotective actions and prevented cardiac dysfunction under various cardiac injurious conditions including ischemia–reperfusion cardiac injury [39] and diabetic cardiomyopathy [40].

The involvement of oxidative stress in the cardio–pathogenic action of ISO has been reported by several preclinical studies. This ISO-induced oxidative stress is triggered by disturbed mitochondrial membrane causing leakage of ROS and other free radicals. as well as catecholamine auto-oxidation [41,42,43]. Consequently, excessive production of oxidative agents, along with impairment of cardiac antioxidants, mediate oxidative damage of myocyte contents manifested by elevated cardiac lipid peroxidation (LPO) and protein carbonyl, as well as decreased activities of antioxidant mechanism, thereby aggravating myocardial damage and cardiac dysfunction [6,41]. In the present study, oxidative stress in hearts of ISO-intoxicated mice was demonstrated by a significant rise in myocardial MDA, protein carbonyl, and NO levels, in addition to a decrease in GSH and antioxidant enzyme activities. This is in line with previous reports indicating that injection of ISO increased myocardial LPO, protein carbonyl, and NO, while decreasing antioxidants [7,44,45,46]. The LPO disrupts the membrane fluidity and permeability in addition to deactivating membrane-bound enzymes, leading to cell injury. Additionally, cell destruction is provoked by the covalently modified proteins, which is exacerbated by permeated LPO-derived aldehydes [47]. Furthermore, oxidative alteration of proteins causes protein breakdown via promoting protein aggregation and/or fragmentation, as well as the inactivation of enzymes and transport proteins, which all end in cell death. Furthermore, the interaction of superoxide anions with NO generates peroxynitrite, a strong oxidant agent that exacerbates oxidative damage by further oxidizing cellular components, resulting in cell death [48].

Therefore, enhancing antioxidant capacity and mitigating oxidative damage are crucial strategies for protecting the myocardium from ISO-induced oxidative injury. In this context, the current study showed that pre-treatment of mice with TAX, the potent antioxidant flavononol, markedly attenuated oxidative stress and boosted the antioxidant defense in the myocardium exposed to ISO. This was evidenced by considerably lower MDA, protein carbonyl, and NO levels, while elevating SOD, CAT, and GSH in cardiac tissues of mice pre-treated with TAX prior to ISO administration. In accordance with our results, several investigations have shown that the cardioprotective action of TAX is associated with reduced oxidative markers and elevated antioxidant components, resulting in ameliorated oxidative injury to the myocardium [39,40,49]. Furthermore, TAX exhibited tissue protective effect against chemically-induced oxidative damage in various experimental models, such as acrylamide-induced heart [21] and kidney injury [50], alcohol [25] and carbon tetrachloride (CCl_4_)-induced liver injury [25], and methanol-induced oxidative optic nerve damage [51].

A substantial amount of evidence demonstrates that enhanced ROS production is a critical factor in the activation of pro-inflammatory and stress signaling pathways, facilitating apoptosis in cardiomyocytes and endothelial cells [11,16,52,53,54]. The ISO-induced ROS production facilitates myocardial inflammation through NF-κB activation and the release of pro-inflammatory mediators, subsequently culminating in cardiac apoptosis and injury [55,56]. Consistent with several studies [11,12,18], hearts of ISO-intoxicated mice showed decreased Bcl-2 expression along with increased NF-κB p65, Bax, and caspase-3 expression, as well as TNF-α and IL-6 levels. Increased pro-inflammatory cytokines, including TNF-α and IL-6, can affect the myocardium directly or indirectly through changes in hemodynamic loading conditions, which trigger additional oxidative stress, myocyte contractility and viability alterations, endothelial and myocardial dysfunction, and myocardial necrosis [6,57,58,59]. The ISO-induced myocardial apoptosis is seemingly trigged by persistent ROS generation that facilitates the dissipation of mitochondrial membrane potential and the release of cytochrome c, leading to the execution phase of caspase-3-dependent apoptotic cell death [11,12,60,61]. Thus, the inflammatory response and consequent apoptosis are considered one of the key therapeutically important components associated with cardiac injury and dysfunction.

In the present study, TAX mitigated NF-κB and decreased TNF-α, and IL-1β in the myocardium of ISO-injected mice, indicating its anti-inflammatory action. Moreover, TAX attenuated ISO-induced myocardial apoptosis, as indicated by the diminished Bax and caspase-3 and enhanced Bcl-2 expressions. Accordingly, TAX prevented cadmium-induced renal inflammation and apoptosis via the suppression of NF-κB, TNF-α, IL-1β, Bax, and caspase-3, and increased Bcl-2 expression [26]. Furthermore, TAX attenuated alcoholic liver disease in mice via mitigation of the NF-κB-enhanced inflammatory response and the regulation of Bax, Bcl-2, and caspase-3 expression in the liver [25]. Moreover, TA was found to inhibit inflammatory response and apoptosis through attenuation of TNF-α, IL-1β, IL-6, Bax, Bcl-2, and caspase-3 in CCl_4_-induced liver injury in mice [25]. Additionally, TAX attenuated myocardial apoptosis through upregulation of Bcl-2 protein expression and downregulation of protein expression levels of Bax, cytochrome c, and caspase-3 and 9 in a rat model of myocardial ischemia/reperfusion injury [23]. It has also been reported that inhibition of the NF-κB signaling pathway by TAX alleviated sepsis-induced acute lung injury in a mouse model of cecal ligation and puncture-induced sepsis [62]. The suppressive effect of TAX on ISO-induced myocardial inflammation and apoptosis appears to be attributable, at least in part, to its potential inhibitory effects on ROS production in the heart.

Since activation of Nrf2/HO-1 signaling plays a key role in protecting against ISO-induced acute myocardial injury [9,15,63], we assessed its involvement in the cardioprotective effect of TAX. Consistent with several studies [11,12,17], our findings showed ISO administration induced downregulation of Nrf2 and HO-1 in the heart. Indeed, Nrf2 is a crucial nuclear transcriptional factor that orchestrates the cellular defense against toxic and oxidative insults through regulation of the expression of an array of genes encoding multiple antioxidant and cytoprotective enzymes [63]. Additionally, Nrf2/HO-1 activation has been shown to regulate the inflammatory responses via suppressing NFκB signaling and upregulating anti-inflammatory cytokines [64,65]. Therefore, therapeutic approaches targeting Nrf2/HO-1 signaling can be considered as a potential therapeutic benefit for the treatment and prevention of CVDs where oxidative stress and inflammation are key factors. This assumption is supported by a previous study where Nrf2 knockdown resulted in increased mortality, decreased antioxidant genes expression in the heart, increased cardiac fibrosis, and apoptosis in mice after pathological pressure overload [66]. In this study, treatment of ISO-intoxicated mice with TAX largely upregulated the Nrf2/HO-1 signaling pathway in the myocardium. In accordance, TAX protected against cadmium-induced oxidative tissue injury, inflammation, and apoptosis in the kidney [26], attenuated oxidative stress-induced cellular damage, and apoptosis in human RPE [24], and prevented lipopolysaccharide (LPS)-induced inflammatory injury in mice [67] by stimulating the Nrf2/HO-1 signaling pathway. Thus, the TAX-mediated Nrf2/HO-1 activation is attributed, at least in part, to its antioxidant and anti-inflammatory properties against ISO-induced cardiac injury.

## 5. Conclusions

This study suggests that TAX can be of significant therapeutic benefit against ISO-induced cardiac injury by ameliorating oxidative stress, inflammatory response, and apoptosis in the heart. These beneficial effects of TAX were associated with increased Nrf2 expression and improved antioxidant defenses. Therefore, TAX could be suggested as a promising new protective strategy against ISO-induced myocardial injury and, perhaps, other CVDs, which undoubtedly deserves further research.

## Data Availability

Data analyzed or generated during this study are included in this manuscript.
